# Cross-training between running and cycling: effects on VO_2_max and running performance—a systematic review and meta-analysis

**DOI:** 10.3389/fspor.2026.1843803

**Published:** 2026-05-25

**Authors:** T. Menges, C. Dindorf, J. Dully, M. Fröhlich

**Affiliations:** 1 Endurance Coach GmbH, Berlin, Germany; 2 Department of Sports Science, RPTU University Kaiserslautern-Landau, Kaiserslautern, Germany

**Keywords:** aerobic fitness, cross-training, cycling, endurance training, performance outcomes, running, training transfer, transfer effects

## Abstract

**Background:**

Cross-training between running and cycling is widely used in endurance sports to maintain aerobic capacity while modulating sport-specific mechanical load. However, the extent to which physiological and performance adaptations transfer bidirectionally between these modalities remains unclear. This systematic review and meta-analysis compared the effects of running-only (run-only) and cycling-only (cyc-only) training interventions to evaluate cross-training transfer to sport-specific VO_2_max and running performance.

**Methods:**

Randomized controlled trials with intervention durations of at least four weeks were included. Studies comparing run-only training with cyc-only or combined running–cycling interventions in trained or recreationally active individuals were eligible (*n* = 7). Outcomes included VO_2_max assessed on the treadmill and cycle ergometer, as well as running performance in time trial (TT) (1 mile, 3,000 m, 5,000 m). Random-effects meta-analyses were performed to estimate pooled effects. Standardized mean differences (Hedges' *g*) were calculated based on change scores (post–pre), and between-study heterogeneity was assessed using Cochran's Q and the *I*^2^ statistic. Sensitivity analyses were conducted to evaluate the robustness of the results.

**Results:**

No statistically significant differences between run-only and cyc-only interventions were observed for any outcome. For treadmill-assessed VO_2_max, cyc-only interventions showed a small, non-significant trend favoring run-only training [Hedges' *g* = −0.32, 95% CI (−0.76, 0.13), *p* = 0.16]. For cycle ergometer–assessed VO_2_max, run-only training resulted in comparable adaptations to cyc-only training, with a small, non-significant trend favoring cycling [Hedges' *g* = −0.34, 95% CI (−0.79, 0.11), *p* = 0.14]. Running performance outcomes demonstrated no meaningful differences between interventions [Hedges' *g* = 0.02, 95% CI (−0.62, 0.66), *p* = 0.88]. Effect estimates varied across studies and outcomes, and all confidence intervals included the null effect, indicating substantial uncertainty regarding the direction of potential effects. The small magnitude of these effects suggests that they are unlikely to translate into meaningful practical differences between training modalities. Variability across studies may be related to differences in participant training status and training dose rather than exercise modality.

**Conclusion:**

Current evidence does not indicate clear improvements or decrements in sport-specific VO_2_max or running performance following cross-training between running and cycling over short to moderate intervention periods. However, these findings should be interpreted with caution due to the limited number of studies, methodological heterogeneity, the use of relatively old training protocols, and the presence of studies with some concerns or high risk of bias, as well as wide confidence intervals crossing zero. Accordingly, the available evidence does not support conclusions regarding the interchangeability of training modalities or the effectiveness of cross-training in maintaining sport-specific performance, but rather indicates that no clear between-group differences were detected.

## 1 Introduction

From a practical point of view, cross-training is often implemented to reduce injury risk and prevent overuse-related interruptions in training (1). Running is associated with high injury rates, with up to 79% of long-distance runners experiencing an injury within a one-year period (1) most commonly due to repetitive impact forces (2, 3). Such injuries frequently lead to partial or complete cessation of running training, resulting in detraining effects and performance declines (4). As a result, runners commonly turn to low-impact cross-training modalities, such as cycling, to maintain aerobic fitness and training intensity while minimizing orthopaedic loading (1).

Beyond injury prevention, cross-training is also used to mitigate the risk of overtraining syndrome (OTS), which can occur during periods of rapidly increased training volume or intensity (5). OTS is associated with physiological and psychological disturbances, including hormonal imbalances, elevated resting heart rate, and mood alterations (6). To reduce the negative effects of excessive sport-specific loading, endurance athletes increasingly incorporate cross-training into their training regimens, with the belief that performance can be maintained or enhanced while reducing injury risk and excessive fatigue (1).

The concept of cross-training emerged in the early 1980s alongside the rise of multi-sport events such as triathlon (7). Early studies reported that performance in a primary sport could be maintained despite reductions in sport-specific training, suggesting potential crossover effects between endurance training modalities (8). These findings contributed to the formulation of the cross-training hypothesis, which proposes that athletes may improve performance in one exercise mode through training in another, despite the principle of specificity (8).

Several studies have demonstrated that non-specific but muscularly similar training modalities can positively influence running performance (4, 9). Loy et al. (10) and Mutton et al. (11) reported significant improvements in running performance following stair-climbing exercise or combined running and cycling training, with effects comparable to run-only training. These findings suggest that cycling, as a non-impact and muscularly similar modality (4, 9), may contribute to enhanced or maintained running performance. Proposed physiological mechanisms include the “lactate sink” hypothesis, which suggests that endurance training of non-running muscle groups may increase their capacity to uptake and metabolize lactate during running, thereby potentially reducing lactate accumulation and improving performance (7, 8).

Despite its widespread application, the effectiveness of cross-training compared to task-specific training remains insufficiently understood. While some studies suggest that cycling and running combinations can maintain or improve performance (11–13), others report limited transfer effects, particularly with respect to maximal oxygen uptake (8, 14, 15), and running economy (5, 16). Moreover, there is a lack of clear evidence-based recommendations regarding how long, how often, and at what intensity cross-training should be performed to maintain or improve performance in the primary sport domain. Therefore, it was hypothesized that cross-training interventions involving running or cycling would maintain VO_2_max and sport-specific performance compared with task-specific training, provided that training frequency, duration, and intensity are matched.

Therefore, the purpose of this systematic review and meta-analysis is to evaluate the evidence on cross-training effects between running and cycling. Specifically, this review examines whether cycling training transfers to running performance and whether running training similarly affects cycling performance. In addition, it aims to identify current knowledge gaps and highlight directions for future research.

## 2 Method

### 2.1 Review protocol and eligibility criteria

This systematic literature review was conducted in accordance with the PRISMA (Preferred Reporting Items for Systematic Reviews and Meta-Analyses) 2020 guidelines and was prospectively registered in the PROSPERO database (CRD420261283267). All methodological steps, including the literature search, study selection, data extraction, and quality assessment, were pre-defined in the registered protocol and consistently applied throughout the review process. The review was guided by a clearly structured PICO (Population, Intervention, Comparison & Outcome) framework (17), which informed both the formulation of the research questions and the development of inclusion and exclusion criteria.

Studies were eligible for inclusion if they were randomized controlled trials (RCTs) published in English or German between 1970 and 9 February 2026 and met the conditions outlined in Table 1.

**Table 1:** PICO structure for review strategy.

PICO	Description
Population (P)	Healthy adults (including untrained individuals) Recreationally active and endurance-trained athletes
Intervention (I)	Cross-training programs or sessions, e.g.: Cycling training for runners Running training for cyclists ≥4 weeks interventions
Comparison (C)	Within-subject comparison: Pre-post changes in physiological and performance outcomes assessed before and after the intervention Between-group comparison: Cross-training interventions versus sport-specific training (running for runners, cycling for cyclists, control group representing usual training)
Outcomes (O)	Physiological parameters: VO_2_max/ VO_2_Peak/ aerobic capacity Heart rate metrics Performance parameters: Running economy (RE) Cycling economy (CE) Time-trial or distance performance

Randomized controlled trials including endurance-trained adults (recreational or competitive) with experience in running and/or cycling, including triathletes, were eligible for inclusion. Studies were required to investigate structured cross-training interventions lasting at least four weeks, such as cycling-based training in runners or running-based training in cyclists. Eligible studies had to report at least one physiological outcome (e.g., VO_2_max or VO_2_peak, heart rate) or performance outcome (e.g., running or cycling economy, time-trial performance, or distance performance), and include either pre–post comparisons or between-group comparisons of cross-training and sport-specific training. Studies were excluded if they met any of the following criteria: (1) intervention duration shorter than four weeks, (2) lack of a specific focus on cross-training effects (e.g., strength-only interventions), (3) use of a no-training or non-exercise control group, (4) non-randomized study designs (including observational studies, case reports, reviews, or animal studies), (5) or absence of relevant physiological or performance outcomes.

To identify eligible studies for this review, a comprehensive database search was conducted across four academic platforms: PubMed, Cochrane Library, ScienceDirect and Springer Nature. These databases were selected to ensure thorough coverage of cross-training effects between running and cycling, as well as their relevance in triathlon.

Each database was searched systematically between 15 August 2025 and 9 February 2026 using tailored search strings based on Boolean operators. The search strategy was developed iteratively by combining keywords derived from the PICO framework and refining the terms through preliminary searches to ensure adequate sensitivity and specificity.

### 2.2 Study selection process

**Figure 1:**
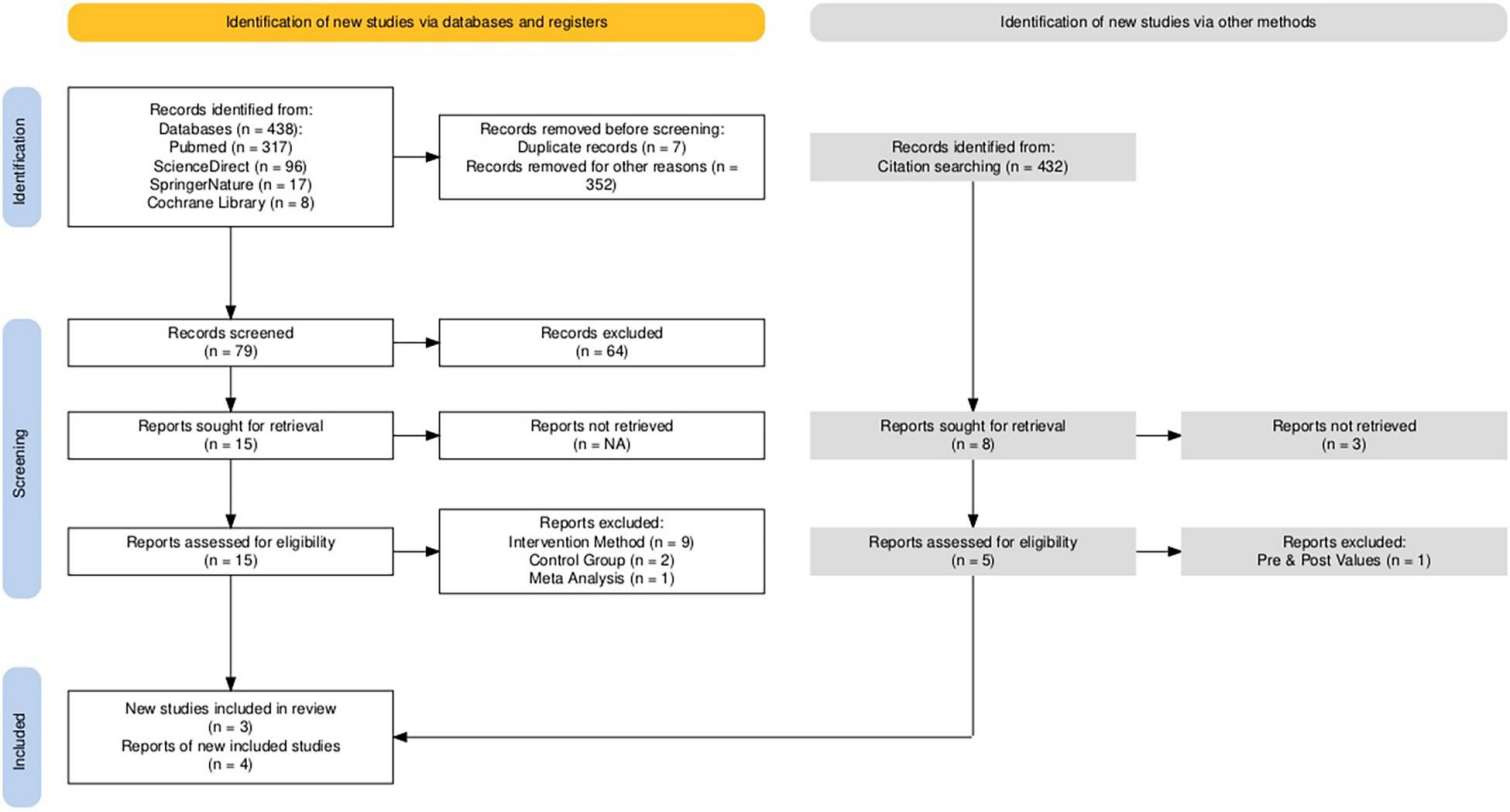
PRISMA flow chart for cross-training. The PRISMA flow diagram was created independently by the authors using the PRISMA2020 Shiny app (41).

The study selection process followed the PRISMA 2020 guidelines and is illustrated in Figure 1. The entire screening and selection process was conducted independently by two independent authors (T.M. & C.D.), with all inclusion and exclusion decisions documented to ensure transparency and reproducibility.

The initial database search identified a total of 438 records across four databases: PubMed (*n* = 317), ScienceDirect (*n* = 96), Springer Nature (*n* = 17), and the Cochrane Library (*n* = 8). Prior to screening, seven duplicate records were removed. Additionally, 352 records were excluded after title and abstract screening due to ineligible study designs (not randomized controlled trials), insufficient sample sizes, lack of pre–post comparisons, absence of reported outcomes, missing control groups, or intervention durations that were too short or because cross-training was defined as non-endurance modalities (resistance or conditioning training).

This resulted in 79 records remaining for title and abstract screening. During the first screening phase, records were assessed for relevance to the target population, intervention type, and outcome measures. As a result, 64 records were excluded, leaving 15 reports for full-text assessment.

Full-text screening led to the exclusion of twelve reports due to predefined exclusion criteria. Specifically, nine studies were excluded because the intervention method did not align with the defined cross-training framework, two studies lacked an appropriate control group, and one study was excluded because it was a meta-analysis rather than an original RCT. Consequently, three studies identified through the database search met all inclusion criteria and were included in the review.

In parallel, additional studies were identified through citation searching (forward and backward snowballing), which yielded 432 records. From these, eight reports were sought for retrieval, of which three could not be retrieved. The remaining five reports were assessed for eligibility, resulting in the exclusion of one study due to insufficient pre- and post-intervention data. Ultimately, four additional studies identified through citation searching were included in the review. In total, seven studies met the eligibility criteria.

### 2.3 Data extraction and management

Data extraction and management were conducted by two independent authors (T.M. & C.M.). All studies meeting the inclusion criteria after full-text screening (*n* = 7) were documented in a structured extraction spreadsheet. Extracted information included study identification (first author, publication year), sample size and participant characteristics, intervention type (cross-training intervention), intervention duration and training frequency, outcome measurement methods, and reported pre- and post-intervention values (means, standard deviations, and sample sizes) for both intervention and control groups.

Data were extracted separately for each outcome domain (VO_2_max treadmill, VO_2_max cycle ergometer, and running performance). Extracted data were subsequently transferred into R (18) for quantitative synthesis.

### 2.4 Risk of bias and quality assessment

The risk of bias of the included studies was assessed in accordance with the PRISMA 2020 guidelines using the Revised Cochrane Risk of Bias tool for randomized trials (RoB 2) (19). The RoB 2 tool was chosen due to its structured, domain-based approach and its suitability for evaluating internal validity in intervention studies examining training effects.

Each study included was assessed independently by two authors across the five RoB 2 domains:

1. Bias arising from the randomization process,
2. Bias due to deviations from intended interventions,
3. Bias due to missing outcome data,
4. Bias in measurement of the outcome,
5. Bias in selection of the reported result

Initial assessments were conducted independently. Inter-rater agreement was quantified using weighted Cohen's *κ* to account for the ordinal structure of RoB 2 judgments (low risk, some concerns, high risk). Following this, discrepancies were discussed and resolved through consensus to derive final domain-level and overall risk-of-bias judgments.

For each domain, judgments were categorized as low risk of bias, some concerns, or high risk of bias, following the RoB 2 signaling questions and decision algorithm. An overall risk of bias judgment was then derived for each study based on the highest level of concern across domains, in accordance with RoB 2 guidance.

In cases where methodological details were insufficiently reported (e.g., unclear randomization procedures, lack of blinding, or incomplete outcome reporting), conservative judgments were applied. All such decisions were documented to ensure reproducibility and transparency, as recommended by PRISMA 2020.

The results of the risk of bias assessment were considered in the interpretation of study findings and informed the narrative synthesis of results. Studies judged to have high risk of bias were not excluded *a priori* but were interpreted with caution in the Results and Discussion sections.

### 2.5 Data synthesis

Data from the included studies were synthesized using meta-analytic methods. A narrative summary was additionally provided to describe study characteristics and to contextualize the quantitative findings. Quantitative synthesis was performed in R using the packages dplyr (20), tidyr (21), and metafor (22) following a standardized procedure to compute standardized mean differences (SMDs) expressed as Hedges *g*, based on change scores (post–pre). All effect size calculations were applied consistently across all included studies.

For each study, mean change was calculated separately for the intervention and control groups between pre- and post-intervention.

To derive the standard deviation of the change score $\left(SD_{\Delta }\right)$, a pre–post correlation of *r* = 0.50 was assumed and incorporated using the following formula (23). This represents a midpoint estimate in the absence of reported correlations. Sensitivity analyses varying r between 0.20 and 0.80 indicated that the pooled effect estimates remained stable across plausible assumptions.

$$SD_{\Delta }=\sqrt{SD_{\mathrm{pre}}^{2}+SD_{\mathrm{post}}^{2}-2r\cdot SD_{\mathrm{pre}}\cdot SD_{\mathrm{post}}}$$

Change scores and their corresponding standard deviations were used to calculate study-level effect sizes (Hedges' *g*) as the difference between intervention and control group changes (INT−CON), together with the corresponding sampling variances required for study weighting.

Meta-analyses were conducted using random-effects models to account for potential methodological and clinical heterogeneity between studies. Between-study variance (*τ*^2^) was estimated using the restricted maximum likelihood (REML) estimator. Statistical heterogeneity was assessed using Cochran's Q and quantified using the *I*^2^ statistic. Results were summarized graphically using forest plots, displaying pooled effect estimates with 95% confidence intervals.

Due to the limited number of available studies for track-based running performance outcomes, cross-training interventions that included cycling (cycling-only and combined running and cycling) were pooled into a single category to enable quantitative synthesis, although this approach may increase heterogeneity.

Analyses were conducted separately for the following outcome domains and comparisons:

1. VO_2_max (treadmill): cyc-only vs. run-only
2. VO_2_max (cycle ergometer): run-only vs. cyc-only
3. Running performance outcome (1 Mile, 3,000 m, and 5,000 m): cyc-only or cycling & running vs. run-only

Potential sources of heterogeneity were addressed by conducting separate meta-analyses for each predefined outcome domain and measurement modality. Sensitivity analyses were conducted by varying the assumed pre–post correlation used to derive change-score standard deviations (*r* = 0.20–0.80) and re-running the random-effects models to examine the stability of pooled effect estimates across plausible assumptions.

## 3 Results

### 3.1 Study characteristics

Seven studies were included in the review, published between 1974 and 2003 and conducted primarily in the United States. The study designs consisted of RCTs. Sample sizes ranged from *N* = 11 to *N* = 60, and participant populations were heterogeneous, including untrained young adults [untrained females (12) and healthy male adults (14, 25, 26)], moderately trained individuals with moderate VO_2_max levels (55.2 mL·kg⁻^1^·min⁻^1^) (11), and trained to competitive runners [cross-country (24) and college distance runners (27)] (Table 2).

**Table 2:** Study characteristics.

Study	Country	Design	Participants	Groups	Exercise Protocol	Main Outcome
Hoffmann et al. (14)	USA	RCT	*n* = 16 healthy male adults	INT: Cycle	9 weeks	VO_2_max (treadmill) (mL·kg⁻^1^·min⁻^1^) improved in both groups (*p* < 0.01):
					(4x/week);	
				CON: Run		
						Run: 56.0 ± 8.09; 66.0 ± 9.0 (17.9%)
					80%–85% max HR	
			(age: 25 ± 4.3 yr; height: 1.80 ± 0.07 m; weight: 79.8 ± 9.8 kg)			Cycle: 55.1 ± 5.37; 63.1 ± 7.81 (14.5%)
						VO_2_max (cycle) (mL·kg⁻^1^·min⁻^1^) improved in both groups (*p* < 0.01):
						Cycle: 50.5 ± 4.95; 58.1 ± 7.10 (15.0%)
						Run: 52.9 ± 6.96; 57.5 ± 7.59 (8.7%)
Mutton et al. (11)	USA	RCT	*n* = 11 moderately fit male runners	INT: Run & Cycle	5 weeks	1 Mile run times (min) improved in both groups (*p* < 0.05):
					(4x/week);	
			(age: 19–35 yr; mean VO_2_max of 55.2 mL·kg⁻^1^·min⁻^1^)			
				CON: Run		Run: 6.3 ± 0.3; 6.0 ± 0.3 (4.8%)
					85%–90% max HR	Run & Cycle: 6.2 ± 0.2; 5.9 ± 0.1 (4.8%)
						5,000 m run times (min) improved in both groups (*p* < 0.01):
						Run: 23.3 ± 1.2; 21.6 ± 1.0 (7.3%)
						Run & Cycle: 22.7 ± 1.1; 21.0 ± 0.6 (7.5%)
						No significant difference between groups.
Paquette et al. (24)	USA	RCT	*n* = 31 cross-country male runners	INT: Cycle	4 weeks	3,000 m run times (sec) improved in both groups (*p* < 0.05):
					(2x/week);	
				CON: Run		
						Run: 706 ± 110; 643 ± 97 (8.9%)
					RPE 11–13	
						Cycle: 757 ± 112; 638 ± 43 (15.7%)
			(age: 15–16 yr; height: 1.70–1.80 m; weight: 55- 65 kg; BMI: 19.1–20.2)			
						Large effect in Cycling (*d* = 1.50)
Pechar et al. (25)	USA	RCT	*n* = 40 healthy young male adults	INT: Cycle	8 weeks	VO_2_max (treadmill) (L/min) improved in both groups significant:
					(3x/week);	
				CON: Run		
						Run (*p* < 0.01): 3.95 ± 383; 4.23 ± 305 (7.1%)
			(age: 19–22 yr; height: 178.1 ± 9.0 cm; weight: 73.3 ± 9.9 kg)		85% max HR	
						Cycle (*p* < 0.05): 4.02 ± 529; 4.13 ± 488 (2.7%)
						VO_2_max (cycle) (L/min) improved in both groups (*p* < 0.01):
						Cycle: 3.50 ± 475; 3.77 ± 414 (7.7%)
						Run: 3.51 ± 283; 3.75 ± 296 (6.8%)
						No significant difference between groups.
Pierce et al. (26)	USA	RCT	*n* = 16 healthy male adults	INT: Cycle	10 weeks	VO_2_peak (treadmill) (mL·kg⁻^1^·min⁻^1^) improved in both groups (*p* < 0.05):
				CON: Run	(4x/week);	Run: 47.8 ± 5.9; 53.5 ± 5.9 (11.9%)
			(age: 31 yr; height: 182.9 ± 7.61 cm; weight: 79.4 ± 10.2 kg)		89% of VO_2_peak	Cycle: 43.6 ± 5.3; 50.4 ± 5.8 (15.5%)
						VO_2_peak (cycle) (mL·kg⁻^1^·min⁻^1^) improved in both groups (*p* < 0.05):
						Cycle: 38.4 ± 4.5; 46.3 ± 5.7 (20.7%)
						Run: 42.6 ± 5.0; 48.3 ± 6.8 (13.3%)
Ruby et al. (12)	USA	RCT	*n* = 18 healthy untrained females	INT1: Run INT2: Cycle	10 weeks	VO_2_max (treadmill) (L/min) improved in both groups (*p* < 0.05):
					(4x/ week);	
						Run: 2.32 ± 0.07; 2.57 ± 0.10 (10.8%)
			(age: 18–25 yr)	CON1: Cycle	70%–80% HR reserve	
						Cycle: 2.32 ± 0.01; 2.49 ± 0.10 (7.3%)
				CON2: Run		
						VO_2_max (cycle) (L/min) improved in both groups (*p* < 0.05):
						Cycle: 2.07 ± 0.09; 2.34 ± 0.09 (13.0%)
						Run: 2.16 ± 0.04; 2.30 ± 0.12 (6.5%)
White et al. (27)	USA	RCT	*n* = 11 female college distance runners	INT: Run & Cycle	5 weeks	3,000 m run times (min) became slower in both groups:
					(7x/week);	
						Run: 10:38 ± 0:20; 10:47 ± 0:37 (−1.4%)
				CON: Run	75%–80%	
					max HR	Run & Cycle: 10:35 ± 0:32; 10:57 ± 0:37 (−3.5%)
			(age: 20.3 ± 1.5 yr; height: 168.9 ± 6.1 cm; weight: 55.2 ± 4.7 kg)			

Across studies, the interventions compared modality-specific endurance training and cross-training modalities, most commonly cycling, running, or combined running and cycling training. Control conditions were modality-specific and depended on the testing modality: run-only training was used as the control condition for treadmill-based VO_2_max comparisons and running performance outcomes, whereas cyc-only training served as the control condition for cycle-ergometer VO_2_max comparisons. Intervention durations were at least four weeks and ranged up to ten weeks, with training frequency typically between three and seven sessions per week, depending on the protocol. Exercise intensity was commonly prescribed using heart rate targets (e.g., relative to maximal heart rate or heart-rate reserve) or relative intensity based on VO_2_max/ VO_2_peak.

Outcomes primarily focused on aerobic capacity and running performance. VO_2_max was assessed using graded exercise protocols on a treadmill and/ or a cycle ergometer, sometimes supplemented by additional physiological measures such as ventilatory threshold, lactate threshold, or running economy. Running performance outcomes included race or time-trial results across distances of 1 Mile, 3,000 m, and 5,000 m, assessed on the track. Key characteristics of the included studies are summarized in Table 2.

Pre- and post-values for the outcome are presented separately in the appendix (Supplementary Appendix Tables 6–8).

### 3.2 Risk of bias in included studies

Inter-rater agreement for the risk-of-bias assessment was substantial (weighted Cohen's *κ* = 0.75), indicating agreement beyond chance (28), with an overall percentage agreement of 88.1%. Discrepancies between raters were infrequent and mainly observed in domain 2 related to deviations from intended interventions and measurement of the outcome, suggesting minor interpretational differences rather than systematic disagreement. Detailed domain-level assessments and overall risk of bias ratings for all included studies are presented in the Appendix (Supplementary Appendix Figure 7). This table provides full transparency regarding methodological quality and potential sources of bias for each study.

Across all included studies, the most frequent limitations were found in the randomization process (Domain 1) and selection of the reported result (Domain 5), as randomization procedures were often insufficiently described and pre-specified analysis plans or trial registrations were generally unavailable. In contrast, measurement of VO_2_max outcomes (Domain 4) was mostly rated as low risk due to objective laboratory-based testing procedures. The domain-level judgments differed between meta-analyses and are summarized below.

#### 3.2.1 Outcome Vo_2_max assessed on treadmill & cycle ergometer

**Figure 2:**
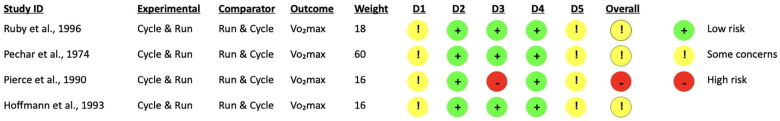
Risk of bias VO_2_max assessed on treadmill & ergometer.

For both meta-analyses (VO_2_max assessed on the treadmill and on the cycle ergometer), the same four studies (12, 14, 25, 26) were included. As the study pool did not differ between comparisons, the risk of bias was assessed jointly using the ROB-2 tool and is discussed in a single consolidated section (Figure 2).

Across studies, the randomization process (D1) raised some concerns, primarily due to insufficient reporting of random sequence generation and allocation concealment. In contrast, deviations from intended interventions (D2) were consistently judged as low risk, indicating that the prescribed training interventions were reported as planned and were comparable in terms of intensity and volume within each study.

The domain missing outcome data (D3) was rated as low risk in most studies; however, Pierce et al. (26) was judged as high risk of bias due to a substantial dropout rate, with more than half of the initially enrolled participants failing to complete the intervention. Reported reasons for attrition included musculoskeletal issues, time constraints, relocation, and personal factors, raising concerns about potential attrition bias and reduced representativeness of the analyzed sample.

Measurement of VO_2_max (D4) was considered low risk across all studies, as outcomes were assessed using objective and standardized exercise testing protocols on both treadmill and cycle ergometer. Selection of the reported result (D5) was consistently judged as some concerns, as none of the included studies provided pre-registered protocols or clearly pre-specified statistical analysis plans, meaning selective reporting cannot be fully excluded.

Overall, while most studies were judged as having some concerns, Pierce et al. (26) was rated as high risk of bias, driven primarily by the high level of missing outcome data. Consequently, the overall risk of bias across studies in both meta-analyses ranged from some concerns to high risk.

#### 3.2.2 Outcome: running performance (track)

For the outcome running performance, three studies were included (11, 24, 27). As in the other comparisons, Domain 1 (randomization process) showed some concerns across all studies due to incomplete reporting of concealment methods. Domain 2 (deviations from intended interventions) was rated as low risk, indicating that training interventions were delivered as planned. However, two studies showed critical limitations and were rated high risk of bias overall. In Paquette et al. (24) missing outcome data (D3) is rated high risk, reflecting substantial exclusions at follow-up. In White et al. (27) missing outcome data (D3) was rated high risk, mainly due to an injury-related dropout resulting in incomplete follow-up performance data in a very small sample, which could plausibly bias the observed effect. Taken together, these issues indicate that the running performance meta-analysis was affected by missing outcome data and methodological limitations in outcome assessment, whereas VO_2_max outcomes were generally assessed under more controlled and standardised testing conditions.

**Figure 3:**
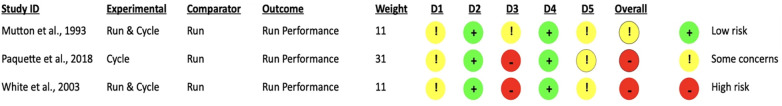
Risk of bias run performance (track): cycling vs. cycling & running vs. running.

Overall, the RoB 2 assessment indicated limitations related to incomplete reporting of randomisation procedures and uncertainty regarding selective outcome reporting. While most VO_2_max outcomes were assessed objectively and showed low risk in outcome measurement domains, running performance outcomes were more vulnerable to missing outcome data and variability in testing conditions. These methodological characteristics are summarised in Figure 3.

### 3.3 Meta analysis result

#### 3.3.1 VO_2_max (treadmill): running vs. cycling

**Figure 4:**
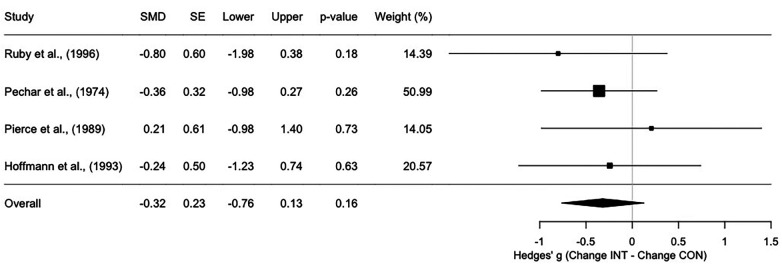
Meta-analysis VO_2_max (treadmill): running vs. cycling.

For the outcome VO_2_max assessed on a treadmill, four studies were included in the meta-analysis (*k* = 4). The intervention group performed cyc-only training, while the control group performed run-only training (Figure 4).

The random-effects meta-analysis showed no statistically significant difference in treadmill-measured VO_2_max change between groups [Hedges' *g* = −0.32, 95% CI (−0.76, 0.13), *p* = 0.16]. The confidence interval included the null effect, indicating uncertainty regarding the presence of a small between-group difference. Between-study heterogeneity estimates were low (*τ*^2^ ≈ 0, *I*^2^ = 0%), and the Cochran Q test was not statistically significant [Q (3) = 1.44, *p* = 0.70]. At the individual study level, effect estimates varied [Ruby et al. (12) *g* = −0.80, Pechar et al. (25): *g* = −0.36, Pierce et al. (26): *g* = 0.21, Hoffmann et al. (14): *g* = −0.24]. All confidence intervals crossed the null effect line (Table 3).

**Table 3:** VO_2_max (treadmill): running vs. cycling.

Study	Change	Change	SD_change	SD_change	Hedges'*g*	Variance
	INT	CON	INT	CON		
Hoffmann et al. (14)	8	10	6.92	8.59	−0.24	0.25
Pechar et al. (25)	0.11	0.27	0.51	0.35	−0.36	0.10
Pierce et al. (26)	6.8	5.5	5.56	5.9	0.21	0.36
Ruby et al., (12)	0.17	0.25	0.09	0.08	−0.80	0.36

#### 3.3.2 VO_2_max (cycle ergometer): cycling vs. running

**Figure 5:**
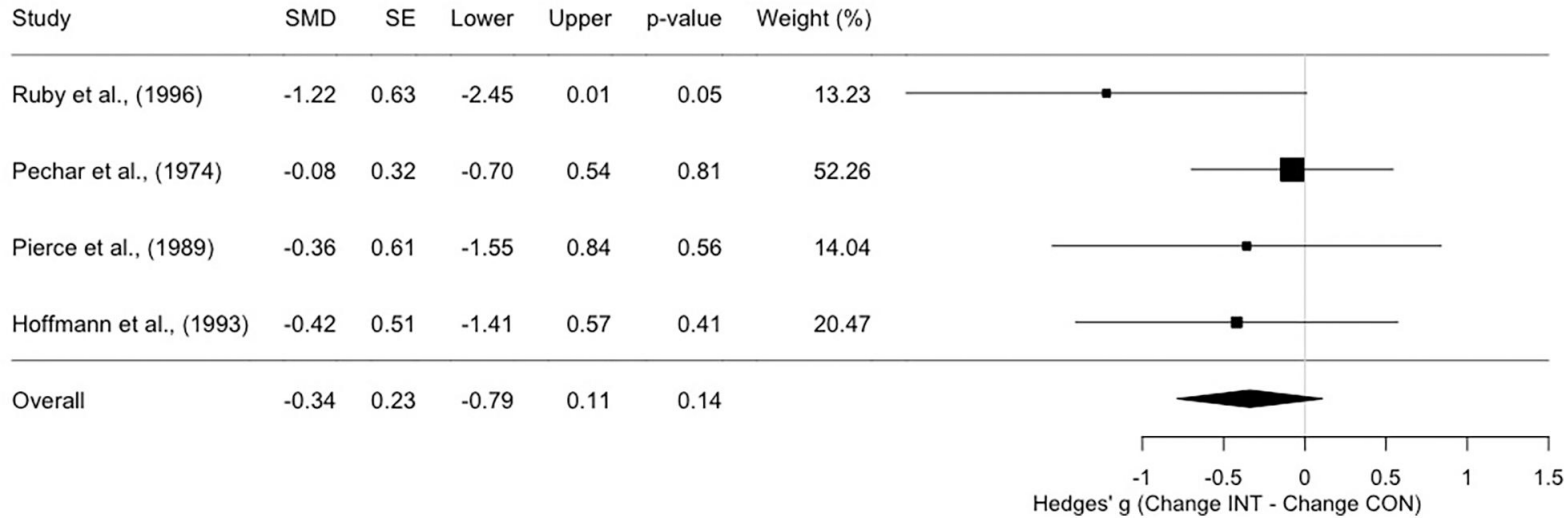
Meta-analysis VO_2_max (ergometer): cycling vs. running.

For the outcome VO_2_max assessed using a cycle ergometer, the same four studies were included in the meta-analysis (*k* = 4). In this comparison, the intervention group performed running only, while the control group performed cycling only (Figure 5).

The random-effects meta-analysis showed no statistically significant difference in cycle ergometer–measured VO_2_max change between groups [Hedges' *g* = −0.34, 95% CI (−0.79, 0.11), *p* = 0.14]. Between-study heterogeneity estimates were low (*τ*^2^ = 0, *I*^2^ = 0%), and the test for heterogeneity was not statistically significant [Q(3) = 2.68, *p* = 0.44]. At the study level, effect estimates were predominantly negative [Ruby et al. (12): *g* = −1.22, Pechar et al. (25): *g* = −0.08, Pierce et al. (26): *g* = −0.36, Hoffmann et al. (14),: *g* = −0.42], with all confidence intervals crossing the null effect line, indicating that no statistically reliable differences were observed between groups (Table 4).

**Table 4:** VO_2_max (cycle ergometer): cycling vs. running.

Study	Change	Change	SD_change	SD_change	Hedges'*g*	Variance
	INT	CON	INT	CON		
Hoffmann et al. (14)	4.6	7.6	7.17	5.74	−0.42	0.25
Pechar et al. (25)	0.24	0.27	0.29	0.44	−0.08	0.10
Pierce et al. (26)	5.7	7.9	6.10	5.20	−0.36	0.37
Ruby et al. (12)	0.14	0.27	0.10	0.09	−1.22	0.39

#### 3.3.3 Running performance (track): race outcomes

**Figure 6:**
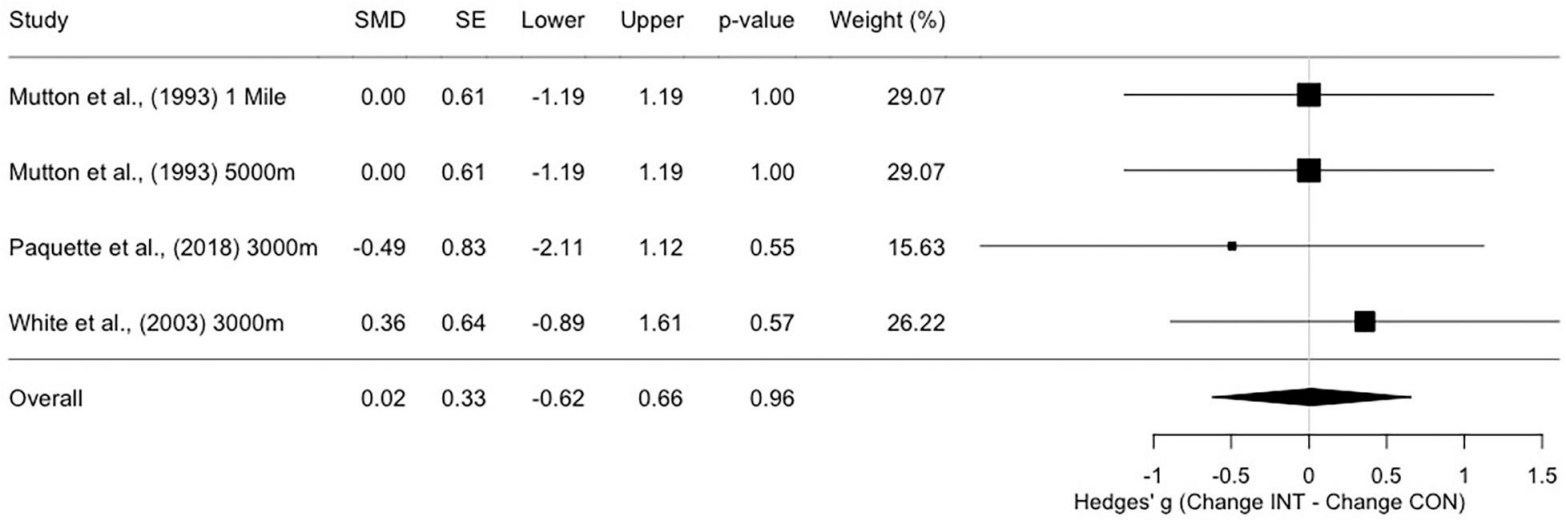
Meta-analysis running performance (track): race outcomes.

For running performance assessed on a track, race outcomes across different distances (1 Mile, 3,000 m, and 5,000 m) were included. In this analysis, the intervention condition consisted of cross-training involving cycling (either cyc-only or combined running & cycling), while the control condition consisted of run-only training (Figure 6). Due to the limited number of available studies, results were pooled across cross-training interventions rather than conducting separate meta-analyses by intervention type.

A total of four comparisons were included in the meta-analysis (*k* = 4). The random-effects model showed no statistically significant difference in race performance outcomes between groups [Hedges' *g* = 0.02, 95% CI (−0.62, 0.66), *p* = 0.88]. Between-study heterogeneity was absent (*τ*^2^ = 0, *I*^2^ = 0%), and the test for heterogeneity was not significant [Q (3) = 0.67, *p* = 0.88]. At the individual comparison level, effect estimates ranged from negative to positive values (Paquette et al. (24) (3,000 m): *g* = −0.49 and White et al. (27) (3,000 m): *g* = 0.36), while two comparisons showed effect estimates of 0.00 [Mutton et al. (11) (1 Mile) and Mutton et al. (11) (5,000 m)]. All confidence intervals crossed the null effect line (Table 5).

**Table 5:** Running performance (track): race outcomes.

Study	Change	Change	SD_change	SD_change	Hedges'*g*	Variance
	INT	CON	INT	CON		
Mutton et al. 1993–1 Mile (11)	−0.3	−0.3	0.17	0.3	0	0.37
Mutton et al. 1993–5,000 (11)	−1.7	−1.7	0.95	1.11	0	0.37
Paquette et al. 2018–3,000 (24)	−1.99	−1.05	1.63	1.73	−0.49	0.68
White et al. 2003–3,000 (27)	0.37	0.15	0.57	0.53	0.36	0.40

## 4 Discussion

### 4.1 Interpretation of the main findings

#### 4.1.1 Overall cross-training effects

Across all meta-analyzed outcomes, including VO_2_max assessed on the treadmill (Hedges' *g* = −0.32), VO_2_max assessed by cycle ergometer (Hedges' *g* = −0.34), and running performance measured on varying conditions (track and grass cross-country course) (Hedges' *g* = 0.02), no statistically significant differences were observed between cyc-only and run-only training groups. Pooled effect estimates across subgroups were consistently small and accompanied by wide confidence intervals crossing zero, did not detect a clear between-group difference in physiological and performance responses between training modalities. However, despite low statistical heterogeneity, substantial conceptual heterogeneity across studies must be considered, including differences in participant training status, intervention structure, testing conditions, and outcome definitions. These differences may limit the comparability of pooled estimates.

Importantly, the direction of individual and pooled effect estimates varied depending on the outcome measure and testing modality, with small trends generally favoring the modality that matched the assessment method. However, these trends were not statistically significant and were inconsistent across studies. This observation should be interpreted cautiously, as it represents an interpretive pattern rather than a formally tested moderator effect. While this pattern may be consistent with the principle of training specificity, which suggests that physiological responses and performance outcomes tend to be highest when assessed in the modality most closely matching the training stimulus, each exercise mode can induce distinct neuromuscular and physiological adaptations (8).

Collectively, the findings indicate that central aerobic capacity, as assessed by VO_2_max, and running performance did not show statistically significant changes over short to moderate intervention periods (≥4 weeks) when cycling-based training was performed either alone or in combination with running. No statistically significant declines were observed across the included comparisons. Evidence from detraining studies suggests that VO_2_max can decline rapidly when endurance training is completely discontinued. For example (29), reported a reduction of approximately 7% in VO_2_max after only 12 days of inactivity. In this context, the absence of significant declines in the present analysis may suggest that replacing part of the running volume with cycling could help attenuate reductions in central aerobic capacity that might otherwise occur during periods without endurance training (30), although this interpretation is indirect, as the present analysis compares active training modalities rather than cross-training vs. complete training cessation. This observation is consistent with the broader concept of cross-training, which has been shown to support general aerobic fitness when different exercise modalities involving large muscle groups are used, particularly in recreational or moderately trained populations (10).

In addition, cross-training programs combining different exercise modalities have been associated with practical benefits such as increased training variety, reduced monotony, and potential injury prevention or rehabilitation support (12, 31). However, given the relatively short intervention durations, the limited evidence base, and the methodological heterogeneity across studies, these findings should be interpreted with caution and do not allow firm conclusions regarding performance enhancement or long-term training equivalence.

#### 4.1.2 Cross-training effects on central aerobic capacity (VO_2_max)

Ruby et al. (12) studied previously untrained adult women over a 10-week intervention period with four training sessions per week at moderate intensity (70%–80% heart rate reserve) and a session duration of 45 min. In this population with large adaptation reserves, treadmill VO_2_max increased in both groups, with a numerically larger improvement in the run-only group compared with the cyc-only group. However, the between-group difference did not reach statistical significance. Given the untrained status of participants and the associated large adaptation potential, these findings should be interpreted with caution, as improvements may primarily reflect general training effects rather than modality-specific adaptations. Previous literature has suggested that training transfer from running to cycling may be greater than vice versa, which may be related to factors such as higher peak heart rates achieved during running, greater mechanical efficiency associated with the stretch–shortening cycle, and differences in pulmonary ventilation and peripheral blood flow in upright locomotion (8, 32, 33).

Pechar et al. (25) included healthy young adults and compared with the other included studies, used a larger sample size (*n* = 20 per group). The intervention lasted eight weeks and consisted of three weekly training sessions at a high relative intensity (85% of maximal heart rate). Although training intensity and volume were matched between groups, treadmill VO_2_max increased significantly in both the running (6.8%; *p* < 0.01) and cycling (2.6%; *p* < 0.05) groups. This improvement was numerically larger following running training; however, the between-group difference did not reach statistical significance. A possible explanation for the minimal improvement observed in the cycling training group may relate to how training intensity was standardized across exercise modalities. However, these interpretations remain speculative, as the study did not directly assess the underlying physiological mechanisms, and the small number of participants limits the robustness of conclusions. In the study by Pechar et al. (25), training intensity was matched using percentages of maximal heart rate obtained from treadmill and cycle ergometer tests. However, similar relative heart rates do not necessarily correspond to identical cardiovascular demands across exercise modes. Running typically involves a larger active muscle mass and can elicit higher stroke volume and cardiac output responses compared with cycling, which may alter the cardiovascular stimulus achieved at a given heart rate (33). Similar methodological considerations were reported by Roberts and Alspaugh (34), who equated training intensity between running and cycling using the PWC150 test. Their results indicated that treadmill training led to notable improvements in cardiorespiratory function when assessed on a cycle ergometer, whereas cycle training resulted in comparatively smaller improvements when evaluated using treadmill testing. In the cycle ergometer test, the running (6.9%) and cycling (7.8%) groups achieved almost identical improvements.

Pierce et al. (26) investigated healthy adult men over a 10-week intervention period with four weekly training sessions performed at intensities above the lactate threshold (89% of pre-training VO_2_peak). Both cycling (15.5%) and running (11.9%) groups showed significant improvements (*p* < 0.05) in treadmill VO_2_peak, and the study reported the only positive effect estimate favoring the cycling intervention. However, this study was characterized by a substantial dropout rate, including withdrawals due to musculoskeletal injuries, which may have influenced the observed effects. The high attrition rate and small final sample size limit the interpretability of these findings and increase the risk of bias. Pierce et al. (26) noted that the higher training frequency, intensity, and caloric expenditure per session (∼500 kcal) compared with Pechar et al. (25) may have induced a strong central cardiovascular stimulus, thereby attenuating modality-specific differences. In the cycle ergometer test, the increases in VO_2_ Peak in the running and cycle groups (13.3% and 20.7%, respectively) were not significantly different.

Finally, Hoffmann et al. (14) reported comparable increases in treadmill VO_2_max following a nine-week intervention in which participants trained either by running or cycling four times per week at progressively increasing intensities, including interval sessions at 90%–95% of maximal heart rate. No significant between-group differences were observed, further supporting the notion that sufficiently high cardiovascular strain can attenuate modality-specific effects on VO_2_max (33).

Taken together, the absence of statistically significant differences across the included studies suggests no clear difference in changes in running-specific VO_2_max between cyc-only and run-only training over short to moderate intervention periods. The small, pooled trend favoring run-only training was not statistically significant and should therefore be interpreted with caution. Differences across studies, including variation in participant training status and training dose, may have contributed to the observed variability in effect estimates.

#### 4.1.3 Cross-training effects on running performance

Currently, no studies have simultaneously examined the effects of run-only and cyc-only training on VO_2_max and running performance, which limits direct comparisons between these outcomes. Moreover, only a small number of studies have investigated the effects of cycling, either alone or in combination with running, on running performance.

Mutton et al. (11) investigated trained adult runners and compared run-only training with combined running and cycling over a five-week intervention period. Performance outcomes at both 1 Mile and 5,000 m showed significant reduced race times but no between-group differences, suggesting that partial substitution of running with cycling did not impair short-distance or longer-distance running performance in trained runners. In addition, VO_2_max responses were assessed on both the treadmill and the cycle ergometer. Both groups showed significant improvements in treadmill VO_2_max (*p* < 0.05) as well as in VO_2_peak measured on the cycle ergometer (*p* < 0.01), with no significant differences observed between the training groups. This finding is consistent with the results reported by Pizza et al. (5), who observed no significant differences in 5,000 m running performance between training modes following an increased training period.

Paquette et al. (24) studied high school cross-country runners at the beginning of the competitive season, replacing two easy runs per week with cycling over a four-week intervention period. No statistically significant between-group differences were observed in 3,000 m performance. However, interpretation of these results is limited by several methodological aspects. Seven runners were excluded from the post-intervention performance analysis, reducing the available sample for the final comparison. In addition, pre- and post-intervention performance tests were conducted under different conditions: the baseline test was performed on a track, whereas the post-intervention test was conducted on a grass cross-country course and took place two weeks after completion of the intervention. These differences in testing environment and timing may have introduced additional variability in performance outcomes and may have affected the comparability of pre- and post-intervention results.

White et al. (27) examined competitive female distance runners during a five-week recuperative phase between seasons, comparing run-only training with a combined running and cycling intervention using a 2:1 cycling-to-running time ratio. One participant withdrew due to an overuse injury, and although post-intervention 3,000 m race times were slightly slower in both groups, no statistically significant between-group differences were observed. Importantly, training intensity was deliberately reduced in both groups (75%–80% of maximal heart rate), and the ratio of running to cycling volume favored cycling, which may explain the uniform performance maintenance across conditions. Similar considerations regarding the relative training load of cycling and running have been proposed in triathlon training models, where cycling training is often prescribed at a higher time volume relative to running to achieve comparable physiological load (35).

Taken together, the absence of significant between-group differences across all included studies indicates that incorporating cycling—either alone or in combination with running—did not show a clear detrimental effect on running performance in the available short-term studies. The small, pooled effect close to zero reflects a balance between studies favoring run-only and those favoring mixed or cycling-based interventions. Still, major methodological flaws have to be considered when interpreting these results. Differences in participant age, training status, intervention timing within the season, training volume distribution, and testing methodology likely contributed more to variability in performance outcomes than the training modality itself. In particular, running performance outcomes differed substantially in terms of distance, testing environment (e.g., track vs. cross-country), and assessment protocols, further limiting direct comparability between studies (36).

### 4.2 Methodological limitations and risk of bias considerations

Interpretation of the present findings is limited by several methodological considerations and substantial heterogeneity across the included studies.

First, sample sizes were small across most trials, with several studies including fewer than 15 participants per group. These small samples reduce statistical power and increase the likelihood that true differences between training modalities were not detected. As a result, non-significant findings should not be interpreted as evidence of equivalence.

Second, participant characteristics varied markedly between studies. Included populations ranged from previously untrained adults (12) to well-trained and competitive runners (11, 27), with differing adaptation reserves and responsiveness to endurance training (32, 37). This variability likely contributed to the observed dispersion in effect estimates and limits the generalizability of pooled results.

Third, the available literature is not only limited in quantity but also predominantly based on training protocols from the 1970s to 1990s. During this period, endurance training programmes were often characterized by relatively uniform training intensities and less structured distribution of training load. In contrast, contemporary endurance training is commonly organized using more systematic intensity distributions and incorporates structured high-intensity interval training sessions aimed at targeting specific physiological determinants of endurance performance (36, 38, 39). Consequently, the training interventions implemented in the included studies may not fully reflect current endurance training practices. This temporal concentration therefore constrains the interpretation of findings and limits their applicability to contemporary endurance training contexts.

Fourth, intervention characteristics differed substantially across studies with respect to duration (4–10 weeks), training frequency (3–5 sessions per week), session duration (20–45 min), and prescribed intensity (70% heart rate reserve to above lactate threshold). These differences in training dose likely exerted a stronger influence on physiological and performance outcomes than training modality alone (40).

Fifth, methodological heterogeneity related to outcome assessment must be considered. Running performance was evaluated using different distances (1 Mile, 3,000 m & 5,000 m) (11, 24, 27), surfaces (grass and track) (11, 24, 27), and testing protocols (24), with one study conducting post-intervention testing two weeks after the intervention period (24) increasing measurement variability. Even in cases where statistical heterogeneity was low, these conceptual differences indicate that pooled estimates should be interpreted with caution, as low *I*^2^ values do not necessarily reflect true comparability between studies.

In addition, publication bias was not formally assessed due to the small number of studies included in each meta-analysis, as methods such as funnel plots or Egger's test are not considered reliable when fewer than 10 studies are available (23). The limited number of included studies further constrains the robustness of the pooled effect estimates and reduces confidence in the precision and generalizability of the findings.

Finally, three studies reported dropouts or exclusions, including withdrawals due to musculoskeletal injuries (26, 27) or exclusion of participants from post-testing (24). These factors introduce additional risk of bias and may have influenced group comparisons.

Taken together, these methodological limitations and sources of heterogeneity indicate that the present findings should be interpreted cautiously and understood as an absence of detected performance decline over short intervention periods, rather than as definitive evidence regarding modality-specific superiority or long-term training equivalence.

### 4.3 Implications for practice and future research

Based on the training protocols used in the included studies, several descriptive characteristics of the interventions can be identified. Across studies, intervention durations lasted a minimum of four weeks and typically involved approximately four training sessions per week (11, 12, 14, 25, 26). Individual training sessions generally lasted around 40–45 min (11, 12), and exercise intensity was prescribed within a moderate to moderately high cardiovascular load, most commonly between approximately 70%–80% and 85%–90% of maximal heart rate or equivalent relative intensity measures (11, 12, 14, 25).

In studies examining running performance, cycling was typically used to replace only a portion of the weekly running volume rather than acting as a complete substitute for running-specific training. Specifically, approximately 20%–50% of weekly running sessions were replaced by cycling-based training (11, 24, 27). In some cases, cycling duration was increased relative to running to achieve comparable cardiovascular or energetic training loads, for example using a 2:1 cycling-to-running time ratio (27).

Under these conditions, no statistically significant declines in VO_2_max or running performance were observed across the included studies. However, these observations should be interpreted cautiously given the limited number of studies, methodological heterogeneity, and variability in study populations and intervention designs.

Future studies should prioritize larger sample sizes, clearer reporting of training intensity and load, and the inclusion of highly trained athlete populations. Additionally, the use of standardized performance tests and modern load quantification approaches would improve comparability across studies. Longitudinal designs examining the effects of prolonged cross-training interventions and their interaction with sport-specific training would further clarify the role of cross-training across different performance levels.

## 5 Conclusion

This systematic review and meta-analysis found no statistically significant differences in VO_2_max or running performance when running and cycling were used to replace one another as training modalities over short to moderate intervention periods. However, these findings should be interpreted with caution given the limited evidence base, the use of relatively old training protocols, the presence of several studies with some concerns or high risk of bias, and wide confidence intervals crossing zero.

Therefore, rather than indicating that the modalities are interchangeable or that cross-training maintains sport-specific performance, the results suggest that no clear between-group differences were detected based on the currently available evidence.
