# Supplementary material for: Cross-training between running and cycling: effects on VO2max and running performance—a systematic review and meta-analysis
Source: Front Sports Act Living. 2026 May 25;8:1843803. doi: 10.3389/fspor.2026.1843803 (PMC13243379; doi:10.3389/fspor.2026.1843803)
Supplement: Supplementary file 2 [file Table2.pdf]

VO$_2$max (ergometer): Cycling vs Running

Table 7: VO$_2$max (ergometer)

Study	group	n	mean_pre	sd_pre	mean_post	sd_post
Hoffmann et al., 1993	INT	8.0	52.9	6.69	57.5	7.58
Hoffmann et al. 1993	CON	8.0	50.5	4.95	58.1	7.10
Pechar et al., 1974	INT	20.0	3.51	0.28	3.75	0.3
Pechar et al., 1974	CON	20.0	3.5	0.48	3.77	0.41
Pierce et al., 1990	INT	5.0	42.6	5.0	48.3	6.8
Pierce et al., 1990	CON	6.0	38.4	4.5	46.3	5.7
Ruby et al., 1996	INT	6.0	2.16	0.04	2.3	0.12
Ruby et al., 1996	CON	6.0	2.07	0.09	2.34	0.09
