# Supplementary material for: Cross-training between running and cycling: effects on VO2max and running performance—a systematic review and meta-analysis
Source: Front Sports Act Living. 2026 May 25;8:1843803. doi: 10.3389/fspor.2026.1843803 (PMC13243379; doi:10.3389/fspor.2026.1843803)
Supplement: Supplementary file 3 [file Table3.pdf]

## Running performance (track)

*Table 8: Running Performance*

| Study | group | n | mean_pre | sd_pre | mean_post | sd_post |
|---|---|---|---|---|---|---|
| Mutton et al., 1993 (1 Mile) | INT | 6.0 | 6.2 | 0.2 | 5.9 | 0.1 |
| Mutton et al., 1993 (1 Mile) | CON | 5.0 | 6.3 | 0.3 | 6.0 | 0.3 |
| Mutton et al., 1993 (5000 m) | INT | 6.0 | 22.70 | 1.10 | 21.00 | 0.60 |
| Mutton et al., 1993 (5000 m) | CON | 5.0 | 23.30 | 1.20 | 21.60 | 1.0 |
| Paquette et al., 2018 (3000 m) | INT | 6.0 | 12.62 | 1.87 | 10.63 | 0.72 |
| Paquette et al., 2018 (3000 m) | CON | 2.0 | 11.77 | 1.83 | 10.72 | 1.62 |
| White et al,. 2003, (3000 m) | INT | 5.0 | 10.58 | 0.53 | 10.95 | 0.61 |
| White et al,. 2003, (3000 m) | CON | 5.0 | 10.63 | 0.33 | 10.78 | 0.61 |
