# Supplementary figures and images for: Cross-training between running and cycling: effects on VO2max and running performance—a systematic review and meta-analysis
Source: Front Sports Act Living. 2026 May 25;8:1843803. doi: 10.3389/fspor.2026.1843803 (PMC13243379; doi:10.3389/fspor.2026.1843803)

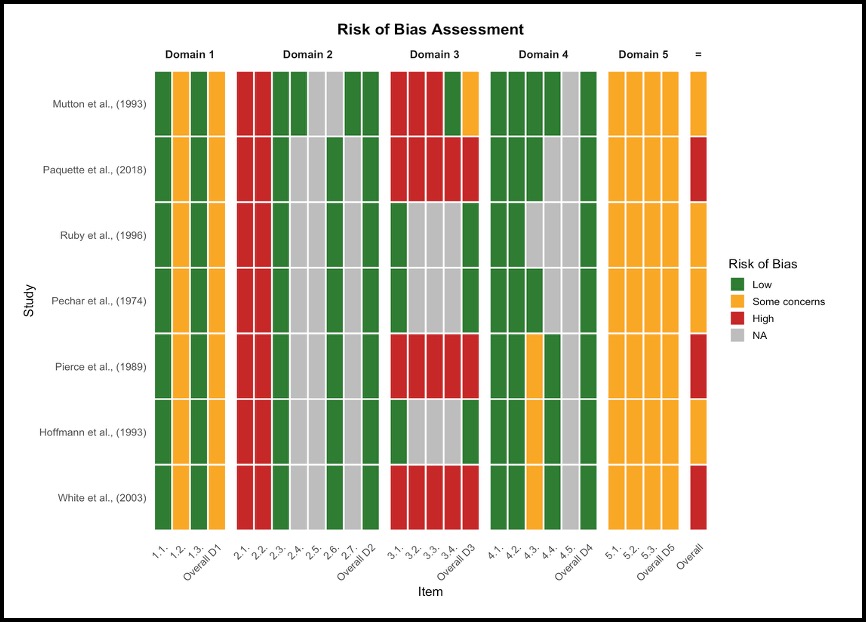

Supplement: Supplementary file 4 [file Image1.jpg]
